# Unraveling the Genome of a High Yielding Colombian Sugarcane Hybrid

**DOI:** 10.3389/fpls.2021.694859

**Published:** 2021-08-13

**Authors:** Jhon Henry Trujillo-Montenegro, María Juliana Rodríguez Cubillos, Cristian Darío Loaiza, Manuel Quintero, Héctor Fabio Espitia-Navarro, Fredy Antonio Salazar Villareal, Carlos Arturo Viveros Valens, Andrés Fernando González Barrios, José De Vega, Jorge Duitama, John J. Riascos

**Affiliations:** ^1^Centro de Investigación de la Caña de Azúcar de Colombia (CENICAÑA), Cali, Colombia; ^2^Research Group in Bioinformatics, Department of Computer Science, Faculty of Engineering, Universidad Del Valle,Cali, Colombia; ^3^Grupo de Diseño de Productos y Procesos, Department of Chemical and Food Engineering, Faculty of Engineering, Universidad de los Andes, Bogotá, Colombia; ^4^Earlham Institute, Norwich Research Park, Norwich, United Kingdom; ^5^Systems and Computing Engineering Department, Universidad de los Andes, Bogotá, Colombia

**Keywords:** sugarcane, genome, assembly, CENICAÑA, CC 01-1940, RNASeq, drought

## Abstract

Recent developments in High Throughput Sequencing (HTS) technologies and bioinformatics, including improved read lengths and genome assemblers allow the reconstruction of complex genomes with unprecedented quality and contiguity. Sugarcane has one of the most complicated genomes among grassess with a haploid length of 1Gbp and a ploidies between 8 and 12. In this work, we present a genome assembly of the Colombian sugarcane hybrid CC 01-1940. Three types of sequencing technologies were combined for this assembly: PacBio long reads, Illumina paired short reads, and Hi-C reads. We achieved a median contig length of 34.94 Mbp and a total genome assembly of 903.2 Mbp. We annotated a total of 63,724 protein coding genes and performed a reconstruction and comparative analysis of the sucrose metabolism pathway. Nucleotide evolution measurements between orthologs with close species suggest that divergence between *Saccharum officinarum* and *Saccharum spontaneum* occurred <2 million years ago. Synteny analysis between CC 01-1940 and the *S. spontaneum* genome confirms the presence of translocation events between the species and a random contribution throughout the entire genome in current sugarcane hybrids. Analysis of RNA-Seq data from leaf and root tissue of contrasting sugarcane genotypes subjected to water stress treatments revealed 17,490 differentially expressed genes, from which 3,633 correspond to genes expressed exclusively in tolerant genotypes. We expect the resources presented here to serve as a source of information to improve the selection processes of new varieties of the breeding programs of sugarcane.

## Introduction

Sugarcane (*Saccharum* spp.) is used worldwide for the production of sugar, bioethanol, and energy (OECD and FAO, 2019), making this crop a main component for the economy of tropical and subtropical countries (Kandel et al., 2018). It is estimated that sugarcane contributes ~80% of world sugar content. In the case of Colombia, the sugar industry has contributed significantly to the economical development of the country and it is considered one of the most efficient sugar industries in the world. Currently, the productivity (Tons of cane / hectare and Tons of sugar / hectare) of Colombian sugar industry is considered the highest in the world, with a total of 12.5 tons of sugar per hectare and 111.7 tons of cane per hectare (Asocaña, 2019). Most sugar cane plantations in Colombia are located along the Cauca river valley with ~238,134 hectares planted in three mega environments: semi-dry, humid and foothill.

Commercial genotypes cultivated today come from a recent domestication process that mainly involves the species *Saccharum officinarum* (x = 10, 2n = 8x = 80) and *Saccharum spontaneum* (x = 8, 2n = 5-16x = 40-128), with possible contributions from haplotypes of species such as *Saccharum sinense, Saccharum Barberi*, and *Saccharum robustum*. The species *S. officinarum* and *S. spontaneum* possess polyploid genomes, although with different chromosomal constitutions (Zhang et al., 2018). Clones of *S. spontaneum* are octoploid (*x* = 8) and possess a number of chromosomes (constitution 2n) that varies between 40 and 128; while *S. officinarum* clones are decaploid (*x* = 10) with a less variable constitution of 80 chromosomes (Price and Daniels, 1968; Sreenivasan et al., 1987). It has been reported that the species *S. spontaneum* conferred characteristics of vigor and resistance, while the species *S. officinarum* provides the high sugar production (Roach, 1972; Sreenivasan et al., 1987). Current hybrids are polyploid with a non-uniform distribution of chromosomes in the same group (aneuploidy), in addition to a highly variable chromosomal constitution (x = 10–13, 2n = 100–130). The genome size of commercial sugarcane hybrids is variable and is estimated to be close to 10 Gbp (Moore et al., 2013).

In addition to its interspecific origin, the genetic complexity of sugarcane is linked to its recent domestication history. Routine genetic improvement of sugarcane dates back to 1888 and was motivated by previous findings showing that this plant was capable of producing viable seed (Moore et al., 2013). The relatively long cultivation cycle, and the consequent low number of selection cycles, has contributed to the maintenance of most of the aneuploidies resulting from interspecific crosses, *S. officinarum* × *S. spontaneum*, in modern hybrids. It is estimated that the genetic content of these chromosomes corresponds to 80% of *S. officinarum*, between 10 and 20% of *S. spontaneum*, and the remaining belonging to recombinant chromosomes of both parents (D'Hont et al., 1996; Piperidis and D'Hont, 2001).

Different strategies have been developed in previous works to reduce the genome complexity and achieve draft genome assemblies. Short Illumina reads, characterized by having a very good quality and an average size ranging from 150 to 500 bp, were initially generated to try to build draft assemblies. However, the short length of these reads generates assemblies with a high degree of fragmentation, even in gene rich regions (SUGESI, 2017). This is because the size of these reads makes it difficult to resolve these types of complex regions within the genome (Jiao et al., 2017), collapsing repetitive and very similar regions within the same assembled fragment (Green, 2002; Treangen and Salzberg, 2011). Advances in long reads sequencing technologies have helped to improve contiguity for other complex genomes, solving repetitive regions with greater precision. Technologies such as PacBio (SMRT) (Eid et al., 2009) or Oxford Nanopore (Branton et al., 2008; Loman et al., 2015) produce average read lengths between 5 and 20 kbp (Thirugnanasambandam et al., 2018). Hybrid assembly approaches have been developed to combine the long range information contained in long reads with the high per base quality of short reads. In addition to long reads, Illumina based protocols such as Hi-C (Lieberman-Aiden et al., 2009) allow to further improve the contiguity of genome assemblies. Hi-C captures DNA-DNA interactions that are produced by the three-dimensional structure of the DNA molecules, providing contiguity information across hundreds of kilobasepairs.

Currently, there are several efforts to sequence both, sugarcane hybrid genomes and the genomes of the parent species *S. spontaneum* and *S. officinarum*. A recent work achieved a high quality genome assembly of the *S. spontaneum* genome (Zhang et al., 2018). This genome was sequenced and assembled from the AP 85-441 genotype, a low heterozygosity tetraploid originating from the SES208 genotype and developed by Moore et al. (1989). *S. spontaneum* has a base chromosome number of 8, and hence, this genotype has a total of 32 chromosomes. A hybrid strategy was used to perform this in this assembly. Sequencing of bacterial artificial chromosome (BAC) libraries was combined with PacBio, Hi-C (Belton et al., 2012) and paired-end Illumina libraries. A chromosome-level assembly was achieved integrating a genetic map developed from an F1 biparental population.

Different breeding programs have sequenced hybrid cultivars of particular interest. This includes the hybrid SP 80-3280, sequenced by Centro Nacional de Pesquisa em Energia e Materiais (CNPEM) and by the University of São Paulo, both located in Brazil (Riaño-Pachón and Mattiello, 2017). In both cases the TruSeq Synthetic Long Read sequencing protocol was used to obtain synthetic long reads (McCoy et al., 2014) but BAC sequencing was included in the second case. This strategy allowed generating an assembly that includes the identification of a total of 373,869 genes, including homologous copies (Souza et al., 2019). The hybrid R570 (Garsmeur et al., 2018) has also been sequenced by a collaboration of different research groups led by Center de Coopération Internationale en Recherche Agronomique pour le Développement (CIRAD). In this case, specific molecules were selected from a BAC library to sequence only gene rich portions of a monoploid genome representation.

One of the most interesting uses of a genome assembly is as information source to aid the reconstruction of metabolic pathways, relating gene annotations to metabolic processes of interest (Oberhardt et al., 2009). The information available in the biochemistry databases can be connected to the genetic information, making it possible to link gene annotations with the corresponding metabolic enzyme that they encode. This simple but powerful union is helpful to predict the set of metabolic reactions catalyzed by the metabolic enzymes of an organism (Edwards and Palsson, 1999; Baart and Martens, 2012; Gu et al., 2019). Enzymes catalyze metabolic reactions configuring packets of information known as metabolic pathways. In this way, it is possible to predict a specific metabolic pathway in an organism based on gene model annotations (Gu et al., 2019). These types of predictions are known as homology-based approximations, because they use existing information from other reported organisms to predict metabolic pathways in organisms that had not been previously studied for metabolism (Gu et al., 2019).

In this manuscript we present the main results of the assembly and downstream analysis of a sugarcane genome from a Colombian hybrid, called CC 01-1940. Using comparative genomics of this assembly with those of close species, we predict functions for a large percentage of the genes annotated in this assembly and we also predict divergence times between the species. Due to the mentioned challenges to achieve genome assemblies in *Saccharum*, information of metabolic pathways is relatively scarce. Hence, a complete reconstruction of the sucrose biosynthesis pathway was predicted from the assembled genome.

## Results

### Genome Assembly of a Commercial Hybrid Sugarcane Variety

**Figure 1:**
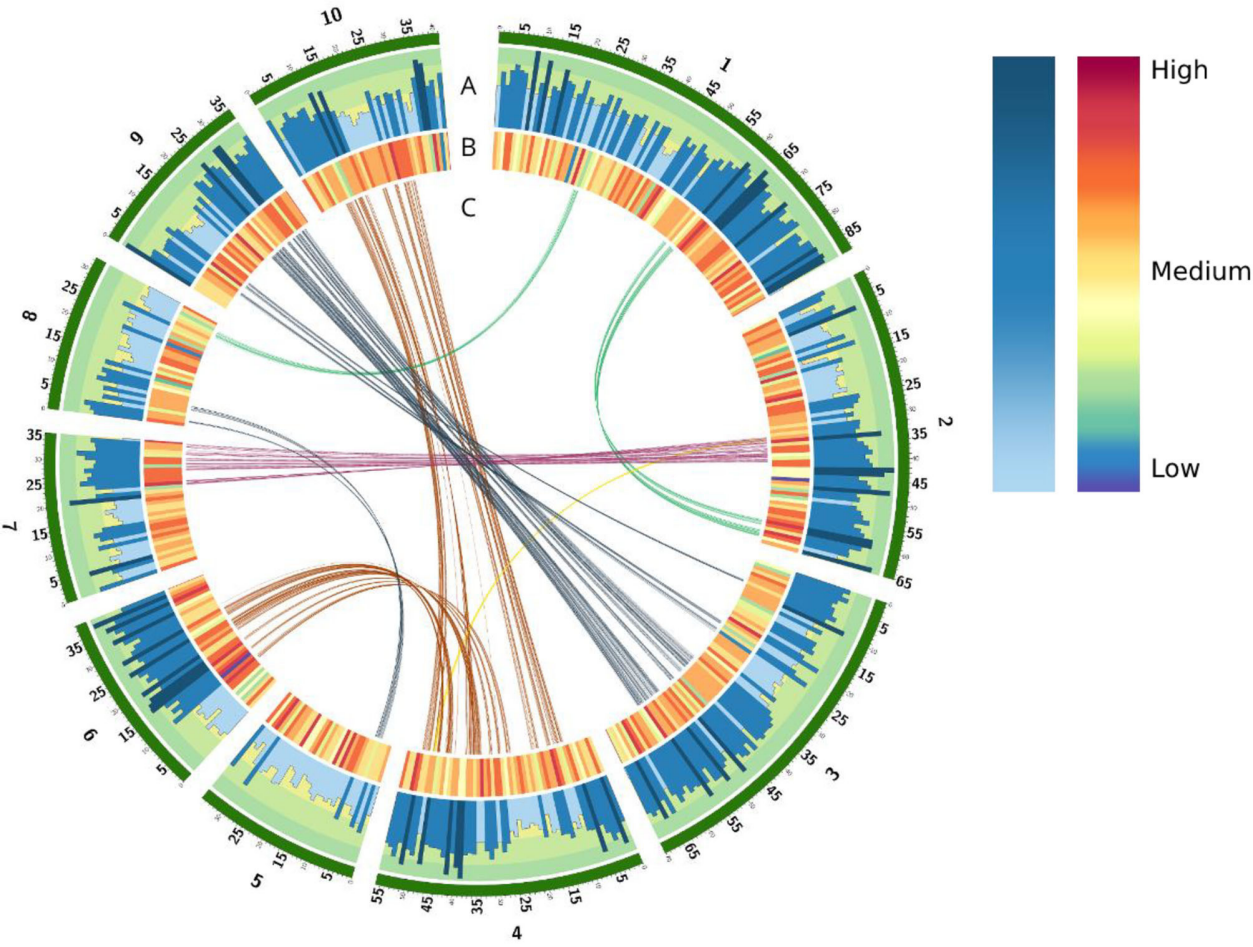
Overview of genomic elements annotated in the CC 01-1940 assembly. In green color are the 10 assembled pseudo-chromosomes. **(A)** Histogram of genes for each chromosome. Each bar represents the total of genes in a windows size of 1 mb. **(B)** Density of TEs in a windows size of 1 mb. **(C)** Duplicate regions in the genome related to whole genome duplication (WGD) events.

We generated a pseudo-chromosome assembly of sugarcane genome from CC 01-1940, a Colombian sugarcane hybrid generated by the breeding program of CENICAÑA. An initial flow cytometry experiment revealed that the CC 01-1940 variety contains approximately a total amount of DNA of 11.21 ±0.374 Gbp and its monoploid genome has a size of 1.019 ±0.031 *Gbp* based on 11*X* ploidy (Supplementary Table 1). Sequencing data from PacBio technology, Illumina short reads and Hi-C reads were combined to achieve a continuity and high quality monoploid genome. From the sequencing process, a total of 357,323,963 paired-end short reads from Illumina were generated with an average length of 2 x 150 bp and an insert length of 360 bp, for a total of 107.2 Gbp of short reads. A total 377,370,721 long PacBio reads were generated, with an average length of 12 Kbp, and a total length of 100.5 Gbp of long reads. Hi-C reads were also generated to perform scaffolding of the contigs assembled from PacBio reads. A total of 496,241,528 paired short reads were generated with an average length of 2 x 150 bp, for a total of 102.87 Gbp of Hi-C reads. An initial draft genome assembly with a median contig length (N50) of 65,986 bp, a total of 42,053 contigs and a total assembly size of 903.1 Mbp was generated running the Flye assembler (Kolmogorov et al., 2020) from the PacBio reads. Using the Hi-C data the ALLHIC Assembler tool (X. Zhang et al., 2019) allowed us to combine 6,967 contigs into 10 groups (Figure 1, green blocks), generating a pseudo-chromosomes assembly with a N50 of 34.94Mbp, a total of 10 pseudo-chromosomes, 44 scaffolds, 35,035 contigs and a total genome assembly of 903.2 Mbp. Considering only the scaffolds included in the 10 pseudo-chromosomes the N50 increases to 55.79 Mbp and the resolved assembly length reduces to 498.3 Mbp.

Base pair level quality was improved by the mapping of Illumina reads to the assembly. A total of 808,524 homozygous sites were corrected, of which 373,543 were SNPs, 384,008 were INDELs and 50,973 were STRs. To assess the base pair level quality conserved plant species orthologs were searched in the assembly using the Busco tool, reaching 96.0% of 425 group of genes known to be conserved in a single copy across plant species (Supplementary Figure 1, Supplementary Table 2). Annotation of repetitive elements was performed using RepeatMasker v 4.1.1 (Smit et al., 2015) and based on a local common sugarcane library of 4,834 transposable elements identified in the CC 01-1940 assembly with RepeatModeler v 2.0 (Smit et al., 2015).

### Genome Annotation

A total of 1,442,953 sequences were identified from the annotation of the repetitive elements generated with RepeatMasker, covering 495.7 Mbp (54.84% of the assembly). A total of 22.67% of the genome was covered by long terminal repeats (LTRs). An additional 2.90% the genome was covered by other class I retrotransposons such as LINE and SINE elements. DNA (Class II) transposons covered the 2.90% of the assembly (Supplementary Table 3). These families of repetitive elements are found homogeneously throughout the entire genome, see Figure 1B.

To perform the structural and functional annotation of gene models for the CC 01-1940 genome assembly, repetitive elements of the assembly were masked, and protein coding genes were annotated combining *de-novo* and reference-based annotation pipelines (see Methods for details). A total of 63,724 gene models were identified with an average gene length of 2,976 bp (Figure 1A), and a total of 68,260 transcripts were predicted with an average coding sequence length of 1,160 bp. The distribution of gene and transcript lengths are consistent with the current annotation of R570 (Garsmeur et al., 2018) and *S. spontaneum* (Zhang et al., 2018) (Supplementary Figure 3 and Supplementary Table 4).

Gene Ontology analysis using the Panther tool (Mi et al., 2019) allowed classifying 31,443 genes within their molecular functions, component cellular and biological processes (Supplementary Figure 2 and Supplementary Table 5). Common ontology terms include response to stress, immune system process, different metabolic processes including sucrose metabolism.

Using the BlobTools tool (Laetsch and Blaxter, 2017) the presence of contamination within the assembly was evaluated (see Supplementary Figure 4). From this analysis it was observed that of the 903.2 Mbp assembled, 901.39 (99.8%) Mbp belong to the streptophytes. This clade or monophyletic group belongs to the Viridiplantae clade, which comprises what is commonly known as green algae and terrestrial plants. Non-plant hits include 0.05 Mbp (0.003%) belonging to the proteobacteria phylum and 0.0016 Mbp (0.00012%) equivalent to 1,688 bases belonging to the Basidiomycota phylum. A total 2.44 Mbp (0.19%) remained without species annotation.

### Comparison With Close Species

**Figure 2:**
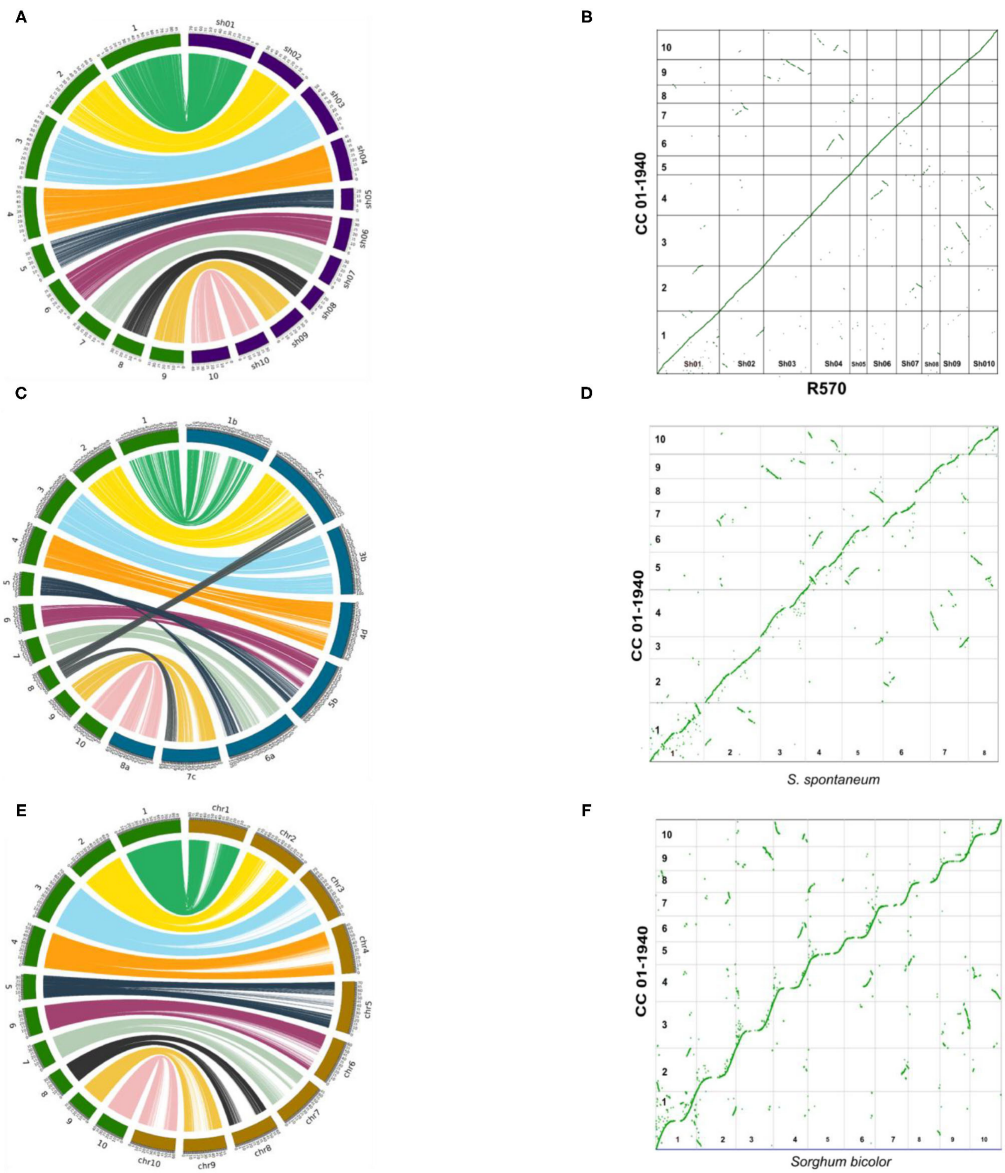
Circus and dot plot graphs showing the collinearity/synteny between the genome of CC 01-1940, and R570 (sugarcane hybrids), *S. spontaneum* and *sorghum bi-color*. In each left graph, CC 01-1940 chromosomes are represented in green, the R570 chromosomes in purple, *S. spontaneum* in blue and *Sorghum* in brown. On the right side, the insertions and translocation between the same genomes are represented using a dot plot graph. **(A)** Collinearity between CC 01-1940 and R570. **(B)** Synteny dot plot between 01-1940 vs. R570. **(C)** Collinearity between CC 01-1940 and *S. spontaneum*. **(D)** Synteny dot plot between 01-1940 vs. *S. spontaneum*. **(E)** Collinearity between CC 01-1940 and Sorghum. **(F)** Synteny dot plot between 01-1940 vs. Sorghum.

Comparative genomics was performed between the genome of CC 01-1940 and recently assembled genomes of sugarcane or close species, including the sugarcane hybrid R570 (Garsmeur et al., 2018), the genome of *S. spontaneum* (Zhang et al., 2018) and the genome of Sorghum (*S. bicolor*) (Paterson et al., 2009). The 10 chromosomes of CC 01-1940 presented high collinearity with chromosomes of R570 (Figure 2A), *S. spontaneum* (Figure 2C) and the Sorghum genome (Figure 2E). A total of 2,850 genes were identified in synteny with the Sorghum genome, distributed in a total of 570 blocks; each block contains an average of 5 genes in synteny with an identity percentage of 91.2% and an e-value 7.9e-07. Compared to the R570 genome, a total of 2,460 genes were identified in synteny, distributed in a total of 492 blocks; each block contains an average of 5 genes in synteny with a percentage of identity of 95.0% and an e- value 4.92006e-07. Compared to the *S. spontaneum* genome, a total of 1,295 genes were identified in synteny, distributed in a total of 259 blocks; each block contains an average of 5 genes in synteny with a percentage of identity of 95.3% and an e- value 7.45938e-07. Duplications of regions near the centromeres toward the telomeres of other chromosomes are observed. For example, in chromosome 1 there is a small region near the telomere of this chromosome, which has been duplicated in the telomere of chromosome 2. Similarly, with chromosomes 4 and 6, chromosomes 4 and 10 and chromosomes 3 and 9 (Figure 2B). Regarding the *S. spontaneum* genome, the 10 chromosomes of CC 01-1940 have a high collinearity with the 8 chromosomes of this genome, mainly toward the regions of their telomeres. It is observed how chromosome 5 has been formed from translocation events of chromosomes 5 and 6 of *S. spontaneum*. In the case of chromosome 8 it was formed from translocation events of chromosomes 2 and 8 of *S. spontaneum*. In the case of chromosome 9, it has a high collinearity with chromosome 7 of *S. spontaneum*. See Figure 2C. These events can be visualized in Figure 2D. With respect to the Sorghum genome, the 10 chromosomes of CC 01-1940 present non-linear collinearity between both genomes, mainly toward their non-repetitive regions, as well as some duplication and inversion events. See Figures 2E,F. Duplication events between chromosomes of CC 01-1940 genome were also observed, (Figure 1C).

**Figure 3:**
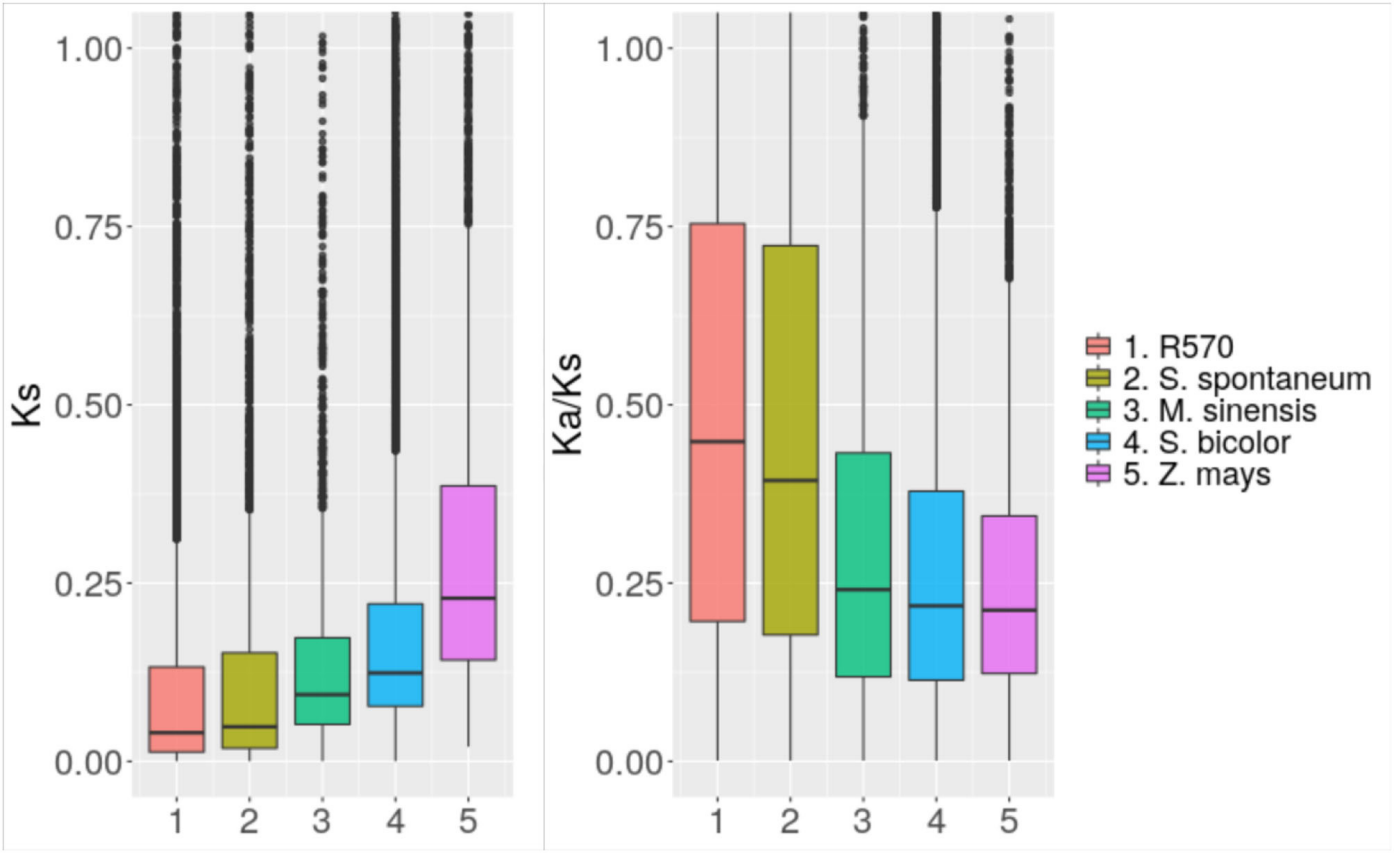
Nucleotide evolution among close species. Distribution of the rate of synonymous mutations (Ks) and the ratio of synonymous to non-synonymous mutations (Ka/Ks) comparing genes annotated in the sugarcane genome assembled in this study with orthologs in the R570 assembly and assemblies of close species.

We performed alignments between the coding sequences of the identified orthologs to calculate rates of nucleotide evolution and identify possible patterns of selection. Figure 3 shows the rate of synonymous mutations per synonymous site (Ks) comparing the nucleotide sequences of synteny orthologs among sugarcane hybrids, *S. spontaneum, M. sinensis, Sorghum bicolor*, and *Z. mays*. Consistent with recent studies on evolution of grasses (Wang et al., 2015), the mode of each distribution was centered at 0, 0.01, 0.03, 0.06, and 0.12 respectively. Taking into account that maize diverged from sorghum about 26 mya, it can be inferred that sugar cane differed from sorghum about 13 mya and from Miscanthus about 6 mya. The divergence between *S. officinarum* and *S. spontaneum* occurred <2 mya.

We also assessed protein evolution through the ratio of non-synonymous to synonymous mutations (Ka/Ks) between orthologs of the different evaluated assemblies. As observed in other species, Ka/Ks values are generally smaller than 1, suggesting purifying selection for most of the genes. Whereas, 85% of the *S. bicolor* synteny orthologs with Ks values above 0.01 showed Ka/Ks values below 0.5, the distribution of this statistic was more uniform between 0 and 1 for synteny orthologs with R570 and *S. spontaneum* respectively, having Ks values above 0.01.

### Reconstruction and Comparative Analysis of Sucrose Metabolism Pathway

**Figure 4:**
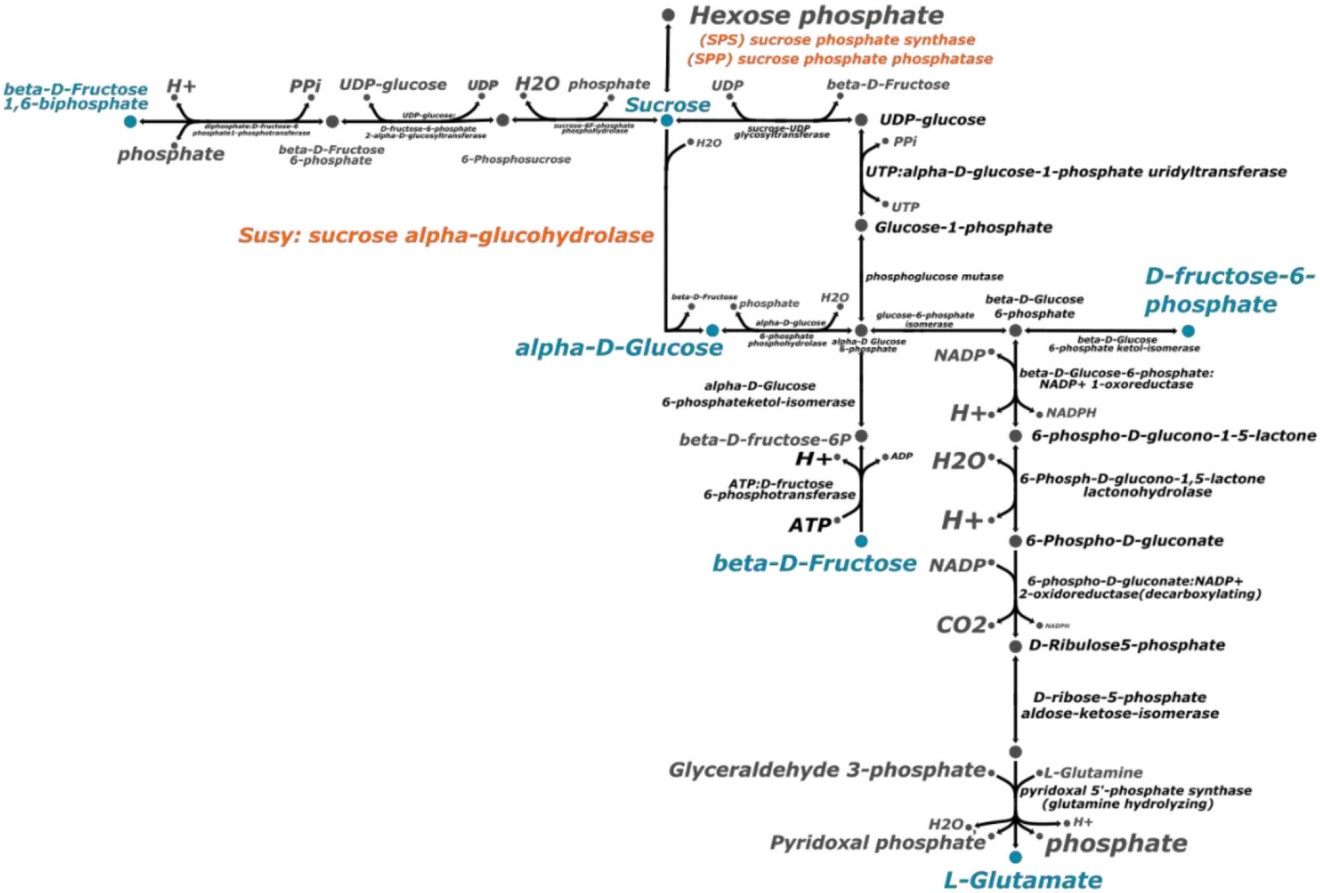
Sucrose biosynthesis metabolic pathway. This metabolic pathway was predicted with the ModelSEED reconstruction server (Seaver et al., 2020). The process to reconstruct the metabolic pathway was repeated with the genome of *Saccharum spontaneum* and *Sorghum bicolor*. The enzymes in orange color are the ones related directly with sucrose biosynthesis. The metabolites highlighted in blue color represent the connection with other metabolic pathways.

Based on the genes annotated in the genome assembly of CC 01-1940, we performed a reconstruction of the sucrose biosynthesis pathway, using the tools for metabolic reconstructions of the ModelSEED database (Seaver et al., 2020). With the information contained in this same database, a total of 19 reactions involved in the synthesis of sucrose were predicted (Figure 4). Among the reactions described, there is one exchange reaction to export sucrose to the extracellular space and 18 synthesis reactions connected with the production of amino acids and glycolysis. The curation of the proposed pathway was performed based on the starch and sucrose consensus metabolic map available in the KEGG database. Enzymes like Invertase (INV) and sucrose synthase (Susy) catalyze sucrose hydrolysis in plants, whereas sucrose phosphate synthase (SPS) and sucrose phosphate phosphatase (SPP) are responsible for sucrose synthesis. These enzymes are regulated at post-transcriptional level in sugarcane into specific signaling pathways (Wang et al., 2017).

Sucrose synthase (SuSy) belongs to the glycosyltransferase-4 subfamily of glycosyltransferases. This is a large family of enzymes that includes Sucrose phosphate synthase (SPS) and trehalose phosphorylase (Stein and Granot, 2019). Sucrose phosphate synthase (SPS) catalyzes the conversion of fructose-6-phosphate and uridine diphosphate-glucose into sucrose-6-phosphate. The activity and localization of these enzymes can be controlled by reversibility phosphorylation process controlled by calcium-dependent kinases (Bilska-Kos et al., 2020). In turn, sucrose-phosphate phosphatase (SPP) catalyzes the final step of the sucrose biosynthesis pathway. Studies on biochemical properties of SPP isoforms are scarce and no specific studies of the completed isoforms of a singular species has been done so far (Albi et al., 2016).

The complete set of specific enzymes have been identified on the assembled genome. The same process of identification was carried out for the species *S. spontaneum* and *Sorghum bicolor*. In both cases the same prediction of reactions and metabolites was obtained.

### Differential Gene Expression and GO Enrichment

We used a dataset comprising 54 Illumina RNA-seq libraries (samples) previously produced by CENICAÑA to identify sugarcane genes associated with tolerance to water stress conditions of drought and flooding (Supplementary Table 6). The dataset corresponds to leaf and root tissue of plants of five sugarcane genotypes characterized by CENICAÑA as contrasting for drought and flooding conditions. Genotypes for the drought condition are CC 93-7711 and SP 71-6949 characterized as efficient in the use of water (tolerant), and MZC 74-275 characterized as not efficient in the use of water (sensitive). Genotypes for the flooding condition are CC 01-1940 and CC 93-4418 characterized as tolerant and sensitive, respectively. RNA-Seq samples come from plants of each genotype under three irrigation treatments for each condition: normal irrigation (control), and mild and severe stress. Drought samples correspond to leaf tissue, while flooding samples correspond to leaf and root tissue.

**Figure 5:**
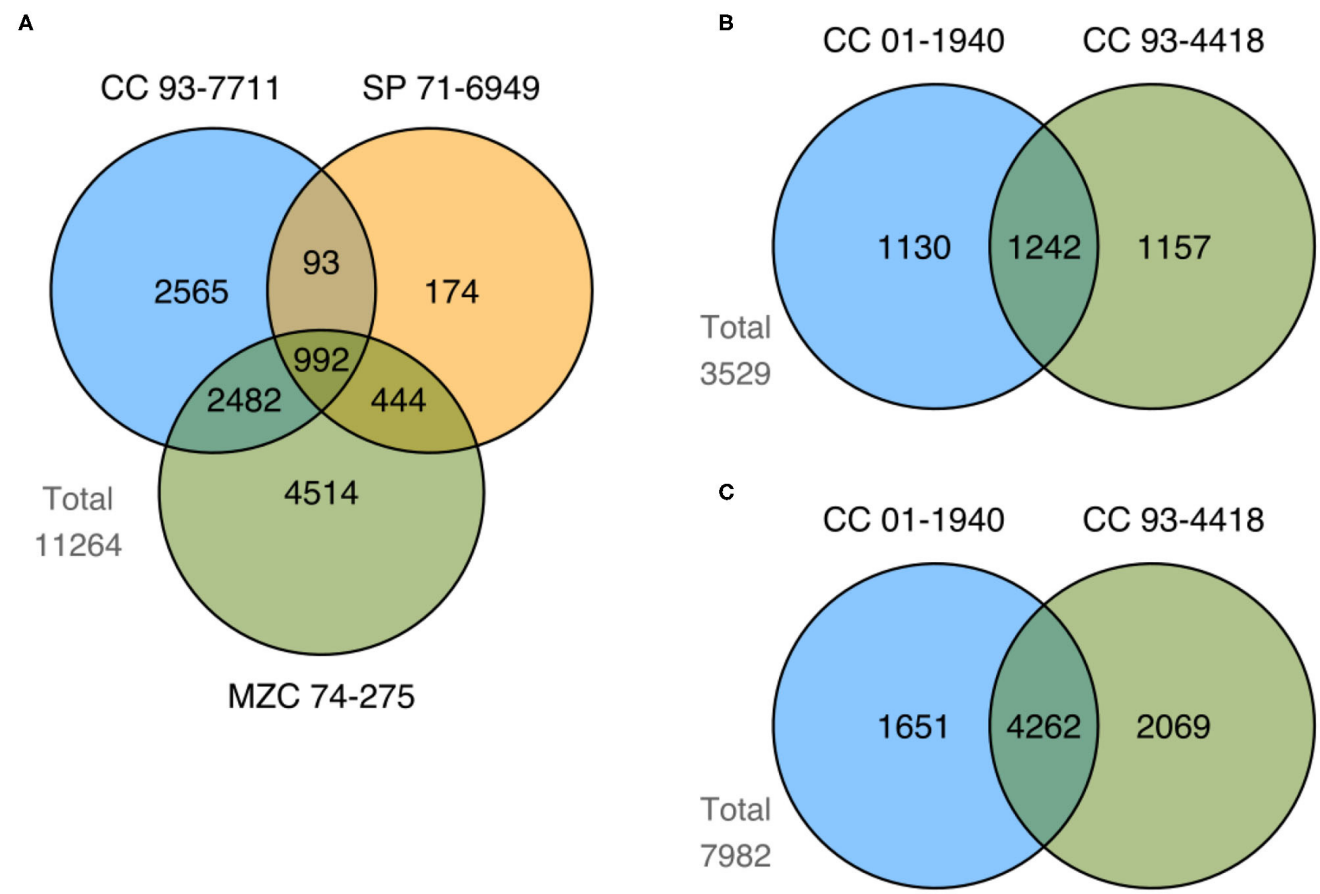
Summary of shared and exclusive DEGs identified in comparisons between control vs. stress treatments of genotypes from both drought and flooding conditions. Figure shows the number of DEGs from tolerant (CC 93-7711 and SP 71-6949) and sensitive (MZC 74-275) genotypes to the drought condition obtained in **(A)** root tissue; and the number of DEGs from tolerant (CC 01-1940) and sensitive (CC 93-4418) genotypes to the flooding condition identified in **(B)** leaf and **(C)** root tissue.

**Figure 6:**
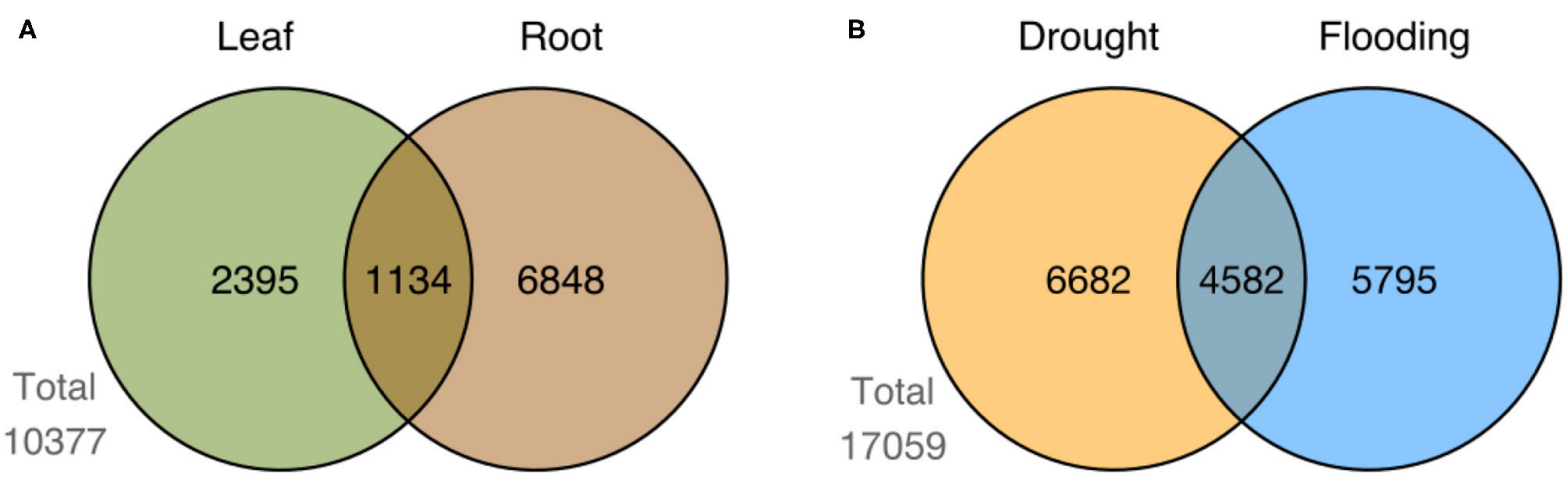
Summary of DEGs by tissue and condition. Figure shows the number of shared and exclusive DEGs between leaf and root tissues in **(A)** flooding, and **(B)** the number of DEGs shared and exclusive to drought and flooding regardless the genotype or tissue.

We identified a total of 17,059 differentially expressed genes (DEGs) by comparing control vs. stress treatment (mild and severe samples unified in a single group) on each available tissue of each genotype for the two studied conditions (drought and flooding). Supplementary Tables 7, 8 show the top 10 most up- and down-regulated DEGs in each genotype for drought and flooding, respectively. We compared sets of DEGs at the genotype level for each tissue and conditioned to find shared and exclusive genes. In the case of drought, we identified 11,264 DEGs in root (Figure 5A). In the case of flooding, we identified 3,529 and 7,982 DEGs in leaf and root, respectively (Figures 5B,C). When comparing sets of DEGs at the level of tissue in flooding, we found 10,377 DEGs, with 1,134 shared DEGs between leaf and root (Figure 6A). We also compared sets of DEGs from the two conditions regardless of tissue and found 12,477 exclusive DEGs and 4,582 shared between drought and flooding (Figure 6B).

### Potential DEGs Contributing to Drought and Flooding Tolerance

We looked for DEGs exclusive to tolerant genotypes (from now on TE-DEGs) as potential genes contributing to tolerance to water stress. We found 2,832 in root for drought, and 1,130 and 1,651 in leaf and root for flooding (Figure 5). Supplementary Tables 9, 10 show the top 30 most up- and down-regulated TE-DEGs for drought and flooding, respectively. Among the TE-DEGs we found homologs to the transcription factor (TF) families AP2/ERF, WRKY, MYB, and NAC. TFs from these families are known for being related to the response to different types of abiotic stress in plant species, including sugarcane, and in some cases as tolerance activators (Jewell et al., 2010; Javed et al., 2020).

In the drought TE-DEGs, we found four homologs to the dehydration-responsive element-binding protein (DREB) family, an AP2/ERF-like (APETALA2/ethylene responsive element-binding factor) TF. DREB TFs have been reported as induced by heat, cold, and salt stress and improved tolerance to drought in several species, including *Arabidopsis*, tobacco, soybean, and sugarcane (Kasuga et al., 2004; Sakuma et al., 2006; Reis et al., 2014; Kidokoro et al., 2015). In the same set of TE-DEGs, we also found 12 homologous sequences to other TFs from the AP2/ERF family, two of which (*cc_00036330*, and *cc_00036331*) are above 80% similar to the *sodERF3* sugarcane gene described as an enhancer of tolerance to salt and drought in tobacco (Trujillo et al., 2008). Regarding the MYB TF family, we identified 19 homologous genes in the drought TE-DEGs set. MYB TFs (present in all eukaryotes) have been observed playing crucial roles in development, disease resistance, and abiotic stress tolerance in plants (Katiyar et al., 2012). For example, soybean and rice MYB TFs have conferred tolerance to chromium, salt, freezing, and drought stress in *Arabidopsis* (Liao et al., 2008; Tiwari et al., 2020). Lastly, three drought TE-DEGs annotated as “Similar to PI21 Protein PYRICULARIA ORYZAE RESISTANCE 21,” are above 85% similar to the sequence of a stress-induced sugarcane gene that conferred tolerance to drought, salt, and oxidative stress in tobacco (Begcy et al., 2012).

In the flooding TE-DEGs, we also found homologous sequences to the AP2/ERF (*n* = 21), MYB (*n* = 22), NAC (*n* = 3), and WRKY (*n* = 19) TF families, and other genes induced under several abiotic stresses, including flooding. WRKY TFs are of particular interest since they are reported to be involved in response to both biotic and abiotic stresses (Phukan et al., 2016). Interestingly, a sunflower WARKY TF increased tolerance to flooding and drought in *Arabidopsis* (Raineri et al., 2015). We identified three homologs to the plasma membrane ATP-ase gene and one homolog to the phenylalanine ammonia-lyase gene. Activity under waterlogging conditions has been reported in soybean and wheat for these two types of genes (Kong et al., 2010; Khatoon et al., 2012). Finally, we found five TE-DEGs homologous to aquaporins, which are known to exhibit activity in plants when subjected to different abiotic stresses and help adapt to flooding stress (Herrera, 2013).

### GO Enrichment of DEGs

We executed a GO enrichment analysis over the sets of TE-DEGs on each condition and tissue. In drought we obtained 96 overrepresented GO terms (BP, MF, and CC categories) in root. In flooding we found 51 and 109 overrepresented GO terms in leaf and root (Supplementary Figure 5). Supplementary Table 11 shows the overrepresented GO terms identified for both drought and flooding conditions. Among the top 20 overrepresented GO terms in the biological process category with the highest number of annotated DEGs (drought and flooding, Supplementary Figures 6A,B), there are 11 different regulation processes, including gene expression, primary metabolism, and nitrogen compound. In the same set, we also identified response to stress and oxidative stress processes in flooding, and the polysaccharide metabolic process in drought which is reported as involved in response to abiotic stress like copper and salt (Liu et al., 2014).

## Discussion

This work describes the efforts developed by our research teams toward a chromosome level assembly of the highly complex sugarcane genome. We improved on the most recent genome available for the hybrid R570, which was focused on reconstructing a gene-rich genome. Taking into consideration the entire contig set the assembled genome length of CC 01-1940 is bigger than that of R570, even when considering the optimized scaffold set that originated the 10 pseudo chromosomes in CC 01-1940. We already have used this genome assembly at CENICAÑA as a resource to align genotyping-by-sequencing (GBS) data of a diversity panel, achieving two times higher mapping rate (79.0 vs. 48.9%) to CC 01-1940, compared to the R570 genome assembly (Manuscript in preparation). This has positive implications for population analyses and mapping of different traits.

We acknowledge that using the synteny with sorghum as part of the assembly process could obscure structural variation events that could modify the current observed collinearity with sorghum. Nevertheless, the chromosome level assembly obtained in this work has been very important for different purposes at CENICAÑA, including comparative genomics, analysis of genotype-by-sequencing and whole genome resequencing data, population diversity estimates, genome-wide association studies and identification of candidate genes and markers necessary to perform molecular breeding schemes for different traits, especially resistance to biotic and abiotic stress. The assembly and annotation of the sugarcane genome reported in this work provided information on the evolutionary history of the *Saccharum* genus, relative to its close ancestors. Considering the relative contribution of *S. officinarum* and *S. spontaneum* in a ratio of about 4:1, the monoploid assembly presented here probably reflects the genome of *S. officinarum*, whereas some of the unplaced contigs could be more related to the contribution of *S. spontaneum*. Based on this premise, the speciation event giving origin to these species was dated <2 mya. Although a parsimony explanation of WGD events would suggest that the two species should share at least one common WGD event, the recent work on the *S. spontenaum* genome (Zhang et al., 2018) predicts a chromosomal reduction before the first WGD in *S. spontaneum*, which is not observed in sugarcane and, consequently, it should not occur in *S. officinarum*. Hence, the octoploid *S. officinarum* genome should be the result of a very recent and independent WGD event. Independent sequencing of the *S. officinarum* genome is needed to completely elucidate the evolutionary history of *Saccharum* species.

The genome assembly of CC 01-1940 not only was useful to make inferences about the evolutionary history of the species, but also to investigate the genetic drivers of important traits such as sucrose synthesis. Sugarcane is a highly productive crop plant with the capacity of storing large amounts of sucrose (Sachdeva et al., 2011). Transport and accumulation of sucrose inside plant tissue have been part of cell models to understand sucrose synthesis. Molecular characterization of genes and transcriptional levels of enzymes involved in sucrose transport and metabolism have been performed in previous studies in other species (Moore, 1995). However, the availability of accurate reference genomes including that of CC 01-1940, provides information to perform a detailed pathway reconstruction based on the information that can be inferred from orthology with other well-studied grass species. Our investigation reveals that genes related to the sucrose synthesis pathway are conserved across grasses, which implies that genetic causes of differences in sucrose storage in sugarcane could be due to single nucleotide polymorphisms or other types of variation altering the expression of key genes. Although different levels of sucrose production are observed under *in vivo* conditions, ortholog conservation indicates that these differences are not produced by changes in the proposed metabolic pathway, but they could be related to differences in the expression levels of genes related to the reactions in the predicted metabolic pathway. Increased expression levels produced by the eight copies of nearly every gene acquired by a recent WGD event could also explain the superior capacity of sugarcane for sucrose storage.

Sucrose is the most important low-molecular weight carbohydrate in most higher plants (Komor, 2000). The sugar originates from photosynthesis that converts inorganic CO2 into carbohydrates. These carbohydrates are stored in forms of polysaccharides such as starch and cellulose. Nevertheless, sugarcane can directly store carbohydrates in the forms of sucrose in stalk parenchymatous cells (Komor, 2000). Sucrose produced in sugarcane has a unique source-sink system. Its stem sinks store the disaccharide in exceptionally high concentrations (close to 650 mM). In contrast, other closer species store starch or cellulose as energy resources with low concentration of sucrose. In addition, sugarcane plants developed the stalk (culm) parenchyma cells as a specialized tissue to accumulate sucrose both inside and outside the cells (symplast and apoplast). Sucrose accumulation in sinks depends on the size and activity of the sinks. This is enhanced with photosynthate production by relieving a feedback repression at metabolomic and transcription level (Wang et al., 2013). Differences between metabolic biosynthesis pathways were not found in our study. Future work includes transcriptional and metabolomic assays to understand the regulation feedback process in sugarcane and elucidate the main difference between dissimilar rate sucrose production between close species (Gantet and Memelink, 2002; Patra et al., 2013).

## Materials and Methods

### PacBio, Illumina Short Reads an HI-C Reads

Sugarcane genomic DNA was extracted from midrib-free tissue from the leaf before the top visible dewlap (TVD) of the accession CC 01-1940 following the protocol proposed by Dellaporta et al. (1983). Plant tissue was disrupted by grinding in liquid nitrogen. The quantification of the extracted DNA, as well as the quality was measured using the Nanodrop 2000 spectrophotometer from Thermo Fisher, while the integrity was corroborated in an 0.8% agarose gel.

Three types of sequencing reads were generated for the assembly, including long Pacific Biosciences (Pacbio) reads, short reads using hi-C sequencing technology to improve continuity within the assembly and Illumina short reads for polishing. Pacific Biosciences (Pacbio) libraries were performed by PacBio RS II and Sequel system sequencing technologies according to the released protocol for both technologies. The PacBio RS II reads were generated at Cold Spring Harbor laboratory (NY, USA) and the Sequel reads at University of Delaware (Newark, DE, USA). High quality short reads were generated using the Illumina technology. Libraries were sequenced on an Illumina HiSeq/MiSeq sequencing technology, using a paired-end 150 run (2 × 150 bases) and insert size of 400 bp. One Hi-C library was generated using the Illumina HiSeq Ten X platform at the company Phase Genomics to produce 150-bp paired-end reads using the restriction enzyme Sau3AI (AAGCTT) (Friedhoff et al., 2001).

### Genome Size Estimation

The genome size, ploidy level and number of potential chromosomes was estimated using a flow cytometry approach. Four samples of leaf tissue, 5 mg of each, were taken from healthy plants to be analyzed using a FACScalibur flow cytometer. For the DNA detection test, an average of 1,000 nuclei stained with propidium iodide were used, following the methodology proposed by Arumuganathan and Earle (1991), fluorescence emission signals (FL2-A) were analyzed using CellQuest software and the mean position of the G0/G1 peak of the nuclei of the sample (M1) and of the internal standard (M2) were determined using the CellQuest software. To estimate the average content of DNA in the nuclei of the plant, calculated values of DNA from nuclei of chicken red blood cells (CRBC), which have a base amount of DNA of 2.33 pg/2C (Galbraith et al., 1983), were used as reference. To estimate the size of the genome (expressed in pg/2C), the ploidy level and the number of chromosomes, previous results calculated for yellow Caledonia were used as a reference, following the methodology proposed by Arumuganathan and Earle (1991).

### Genome Assembly

A chromosome level assembly of the CC 01-1940 was generated by combining the three different next generation sequencing technologies. As a first step, two de-novo assemblers, Canu version 1.7 (Koren et al., 2017) and Flye version 2.8 (Chaisson et al., 2009) were tried starting from 100.5 Gbp of Pacific Biosciences raw reads, which represents about 100 × of the monoploid genome size. The best results were obtained with the Fly assembler. As the second step, a pseudo-chromosomes genome assembly was generated using a total of 107.2 Gbp of Hi-C data with the ALLHIC Assembler tool (Zhang et al., 2019). ALLHIC allows generating a chromosomal assembly for allopolyplod/polyploid genomes, separating each allele, using HI-C paired-end reads. However, for this genome, it was possible to identify a unique haplotype for each allele, generating a monoploid representation. To improve the ordering between the contigs grouped by ALLHIC, synteny analysis with the sorghum genome and the DAGChainer tool (Haas et al., 2004) were performed. This allows us to confirm the orientation of the ordering of the contigs by each group. As the third step, to reduce the error presence in the assembly, a polishing was performed to achieve high base pair quality integration of the 100.7 Gbp of paired-end Illumina Hi-C data to correct base pairs and indels errors. These data were mapped to the assembly using the Bowtie2 (Langmead and Salzberg, 2012) tool, and tandem repeats set from the assembly were identified using the TRF tool (Benson, 1999). These tandem sequences and the mapping file were used in conjunction with the NGSEP tool to identify the variants present between the Illumina reads and the pseudo-chromosomes assembly. Then, these variants were filtered to select only homozygous variants within a VCF file (Danecek et al., 2011). With the VCF file and using the parameter “ReferenceBuilderFromVCF” of NGSEP, a polished assembly was generated where the variants that presented sequencing errors with respect to the generated alignments were corrected.

### Genome Annotation

Annotation of repetitive elements was performed using RepeatMasker v4.1.1. (Smit et al., 2015) The genome assembly was masked by replacing the repetitive regions with N characters to perform annotation. The masked genome was used into two strategies implemented to generate the annotation of the genome. Additionally, the annotation of this genome is presented, which was carried out using the methodologies of MAKER (Cantarel et al., 2008) and Tuxedo (Trapnell et al., 2012), with which we identified the structures and putative functions of the genes present within this assembly.

The first genome annotation strategy combined a structural annotation performed with Tuxedo (Trapnell et al., 2012) pipeline, which uses raw sugarcane RNA-seq data obtained from publications (Santa Brigida et al., 2016; Hoang et al., 2017a,b; Li et al., 2017; Nakayama et al., 2017; Liu et al., 2018), and RNA-Seq data produced by CENICAÑA. This raw data was mapped to the pseudo-chromosomes genome assembly using the Hitsat2 tool (Kim et al., 2019) and the resulting alignment was used with the StringTie tool (Pertea et al., 2015) to assemble and merge the transcripts. Finally, using the “GTF2GFF3Converter” option of NGSEP, the assembly's structural annotation was generated in GFF3 format.

A second genome annotation was performed following the Maker (Cantarel et al., 2008) pipeline v 2.31.9 which included evidence from the CENICAÑA sugarcane transcriptome generated with Illumina Short reads. Additionally, cDNA sequences and protein homology evidence from the R570 genome (Garsmeur et al., 2018)*, S. spontaneum* (Zhang et al., 2018), and ab initio predictions using the AUGUSTUS tool (Keller et al., 2011) were also included to define a confident gene model. Finally, the two annotations were merged using a custom script available within NGSEP (class.ngsep.transcritpme.io.GFFCombineAnnotatios).

Using the merged annotation, the completeness of the assembly was evaluated using the Busco tool v 4.0.6 (Simão et al., 2015). This tool gives a quantitative measure of how complete the genome and its generated annotation is, taking into account the evolutionary information expected in its gene content. In this case, the “Viridiplantae_odb10” lineage dataset was used as a reference, which is made up of 425 groups, made up of 57 plant species (the version of Busco 3 indicates that this database consists of a total of 1,440 genes).

The gene models and transcripts generated for the assembly of CC 01-1940 were analyzed using the NGSEP tool with the parameter “TranscriptomeAnalyzer.” This parameter uses the annotation of a transcriptome in GFF3 format, delivering the statistics of the transcriptome generated from the assembly, including information regarding cDNA, CDS and proteins. Other values such as the number of exons, introns, mRNAs, CDS, percentage of genome covered were calculated using the GAG tools (Geib et al., 2018).

Functional annotation was performed following the Maker pipeline, aligning the proteins sequences against the Uniprot/Sprot database using the BLASTp tool (Mahram and Herbordt, 2010) and selecting only the results that had an E-value below of 1e-10. Subsequently, the InterProScan database was used to identify the protein domains using the InterProScan tool command line and results were classified into the three groups of ontologies: molecular function, biological process and cellular component using the online tool “PantherDb” (http://pantherdb.org) (Mi et al., 2019). The putative gene function and the protein domain information was added to the final annotation in GFF3 format using the MAKER Accessory Scripts.

Annotation of Transposable Elements (TEs) was performed using the RepeatModeler (Smit et al., 2015) integrated with the RECON v 1.08 and RepeatScout v 10.5 tools for the identification of Novo transposable elements (Ts) in the genome and to group the TEs by families. The identified Ts were imported into the RepeatMasker tool (version 4.05) (Smit et al., 2015) to identify, classify, and mask the Ts. TEs that failed to be classified can be classified using the TEclass tool (version 2.1.3) (Abrusán et al., 2009).

### Metabolic Reconstruction of Sucrose Biosynthesis Pathway

ModelSEED metabolic pathway reconstruction tools were used to identify the enzymes involved in the sucrose metabolic pathway (Seaver et al., 2020). A genome scale metabolic model was reconstructed to identify the complete sucrose biosynthesis pathway. The used platform creates new reconstructions annotating plant genomes or predicted proteins with the PlantSEED platform (Seaver et al., 2018). This step is necessary because SEED functional annotations generated by PlantSEED are linked directly to the biochemical reactions in the ModelSEED biochemistry database. Only the information about sucrose biosynthesis pathway was taken into account in this study. The obtained predicted pathway was compared with the information reported in literature about it. The metabolic pathway was diagrammed with Escher (King et al., 2015), it is an online resource to construct metabolic maps using a genome scale metabolic model. The final curated map was used to compare differences between *Saccharum spontaneum, Sorghum bicolor* and the species reported in this study.

### RNA-Seq Data and Differential Gene Expression Analysis

A total of 54 Illumina paired-end RNA-seq (2x100 bp) samples previously produced by CENICAÑA were used in this study. RNA-seq samples correspond to leaf and root tissue of plants of five contrasting genotypes for drought (CC 93-7711, SP 71-6949, and MZC 74-275), and flooding conditions (CC 01-1940, and CC 93-4418). The drought condition comprises samples from leaf tissue, while the flooding condition comprises samples of leaf and root tissue. To produce RNA-seq libraries, plants of each genotype were subjected to three treatments of drought and flooding conditions: (1) control (normal irrigation); (2) mild stress (25% usable water in the soil for drought; 2 days of waterlogging for flooding); and (3) severe stress (no moisture in the soil and permanent wilting of plants for drought; 14 days of waterlogging for flooding). Each sample representing a combination of condition-genotype-tissue-treatment has at least two and up to four biological replicates (Supplementary Table 6). For each condition-genotype-tissue combination, samples from severe and mild stress treatments were unified into a single group called stress to be compared to the corresponding control group. By unifying samples, the number of replicates in the compared groups (control and stress) was between three and four. The total number of reads in the dataset is 3.25 billion, with sample size varying between 16,099,262 and 193,887,078 reads.

RNA-Seq reads were aligned to the genome assembly reported above, using the HISAT2 (v2.1.0) (Kim et al., 2019) software with default options. Read alignments were processed with PICARD Tools (v 2.25.0) (Broad Institute, 2019) to remove duplicated reads coming from PCR artifacts. To assemble and quantify transcripts, deduplicated read alignments were processed with StringTie (v2.1.4) (Pertea et al., 2015) using the genome annotation (-G option) previously generated, and only transcripts in the reference were assembled and reported (-e option). To identify differentially expressed genes (DEGs) in each condition-genotype-tissue combination, the control sample group was compared against its corresponding stress sample group. DEGs were identified with DESeq2 (v1.30.1) (Love et al., 2014), and significant DEGs were selected using thresholds of FDR < 0.05 and log_2_(FC) ± 1. Finally, over-represented GO terms associated with DEGs were identified with an enrichment analysis using the Fisher's exact test (*p*-value < 0.01) executed with topGO (v2.42.0) (Alexa and Rahnenfuhrer, 2010).

## Data Availability Statement

The original contributions presented in the study are publicly available. This data can be found here: NCBI repository, accession number: PRJNA713858 (https://www.ncbi.nlm.nih.gov/bioproject/713858).

## Author Contributions

JT, JR, and JD conceived the study. CL and JD contributed to the assembly of CC 01-1940. MR and AG carried out the construction of the sucrose metabolic pathway. MQ carried out the flow cytometric analysis. HE-N, JR, JD, and CL contributed to the RNASeq project. CV and FS created CC 01-1940. JT, JR, JD, MR, and HE-N drafted the manuscript. All authors read and approved the final version of the manuscript.

## Conflict of Interest

The authors declare that the research was conducted in the absence of any commercial or financial relationships that could be construed as a potential conflict of interest.

## Publisher's Note

All claims expressed in this article are solely those of the authors and do not necessarily represent those of their affiliated organizations, or those of the publisher, the editors and the reviewers. Any product that may be evaluated in this article, or claim that may be made by its manufacturer, is not guaranteed or endorsed by the publisher.
