# Supplementary figures and images for: Unraveling the Genome of a High Yielding Colombian Sugarcane Hybrid
Source: Front Plant Sci. 2021 Aug 13;12:694859. doi: 10.3389/fpls.2021.694859 (PMC8414525; doi:10.3389/fpls.2021.694859)

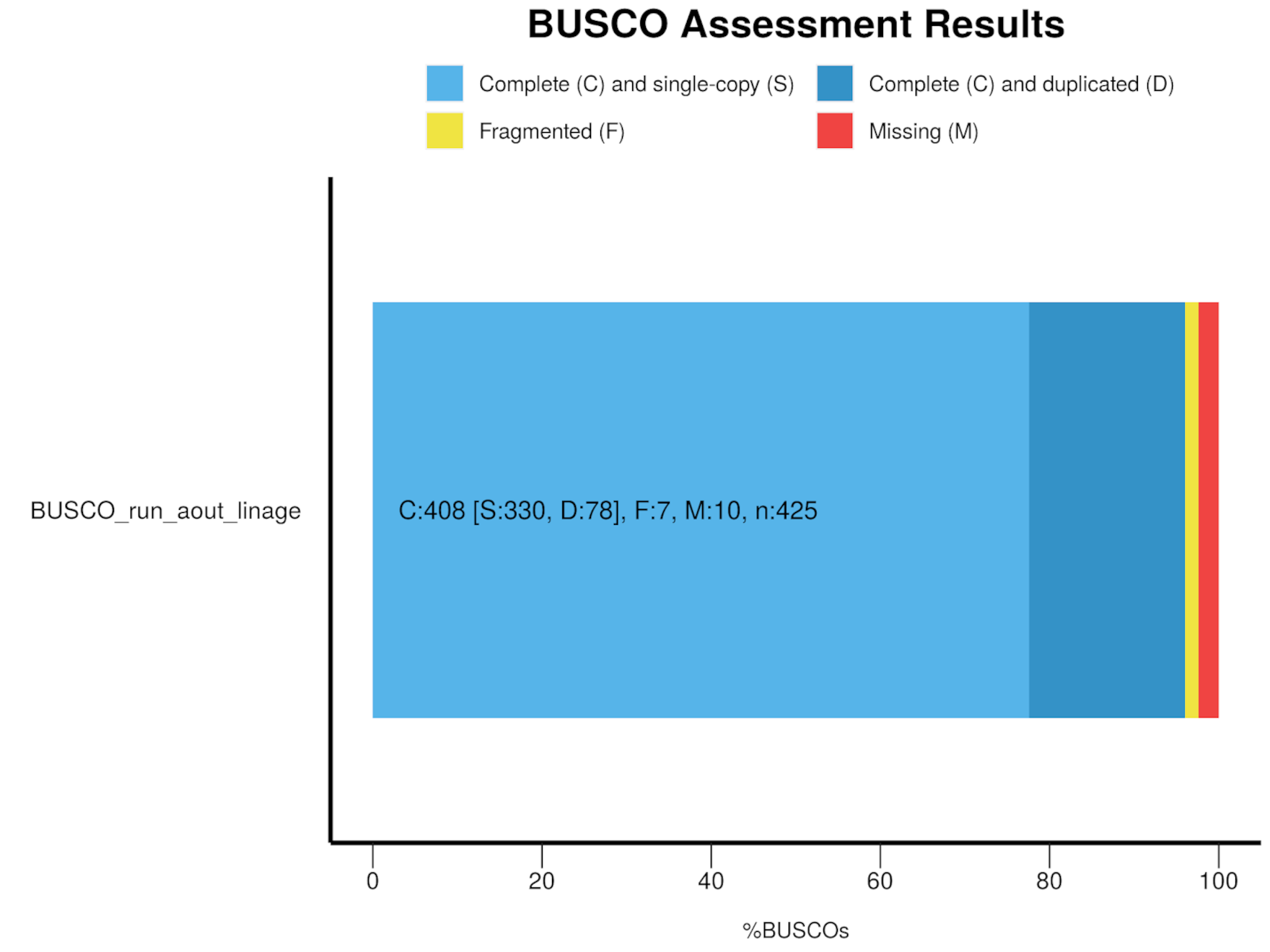

Supplement: Supplementary file 12 [file Image_1.TIF]

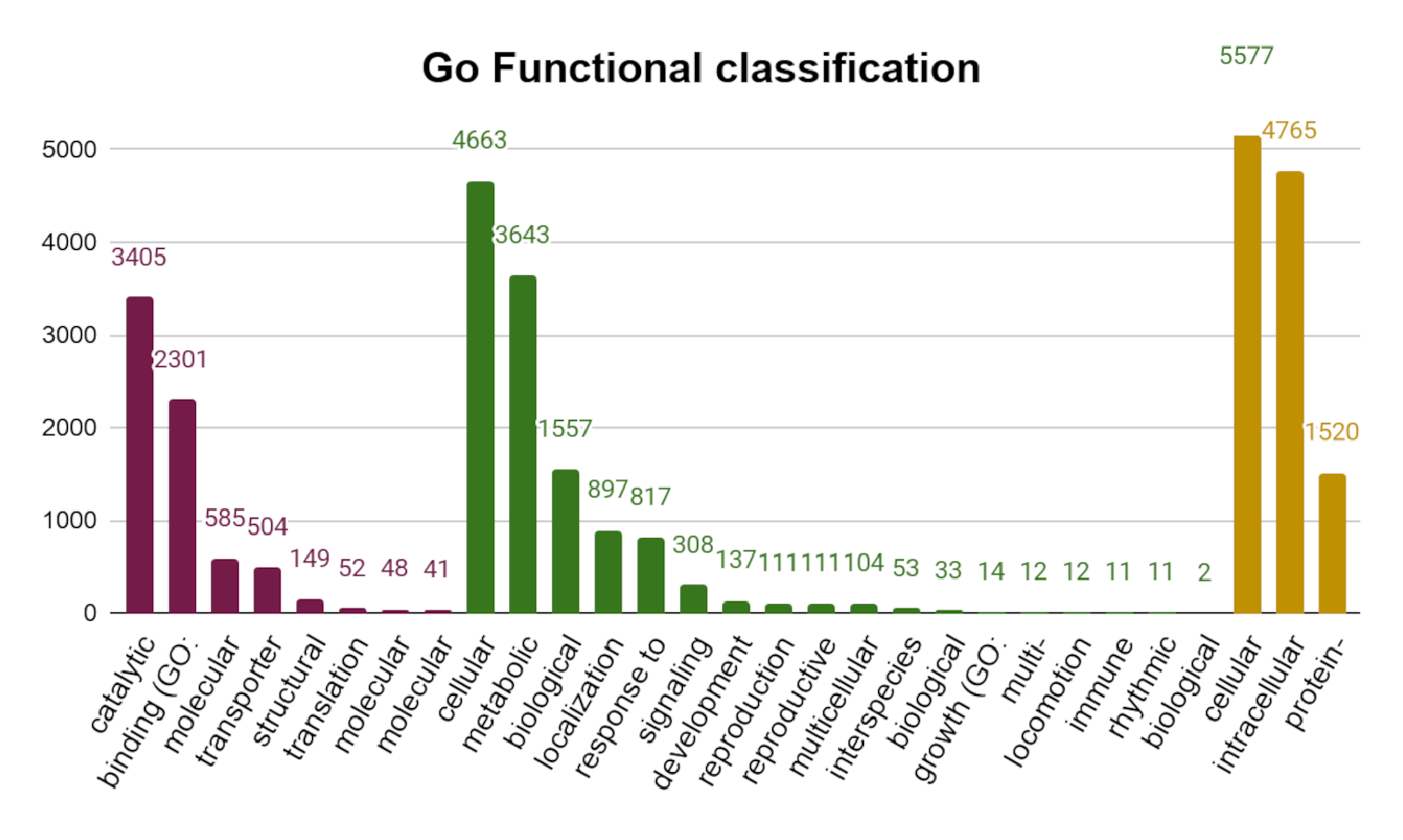

Supplement: Supplementary file 13 [file Image_2.TIF]

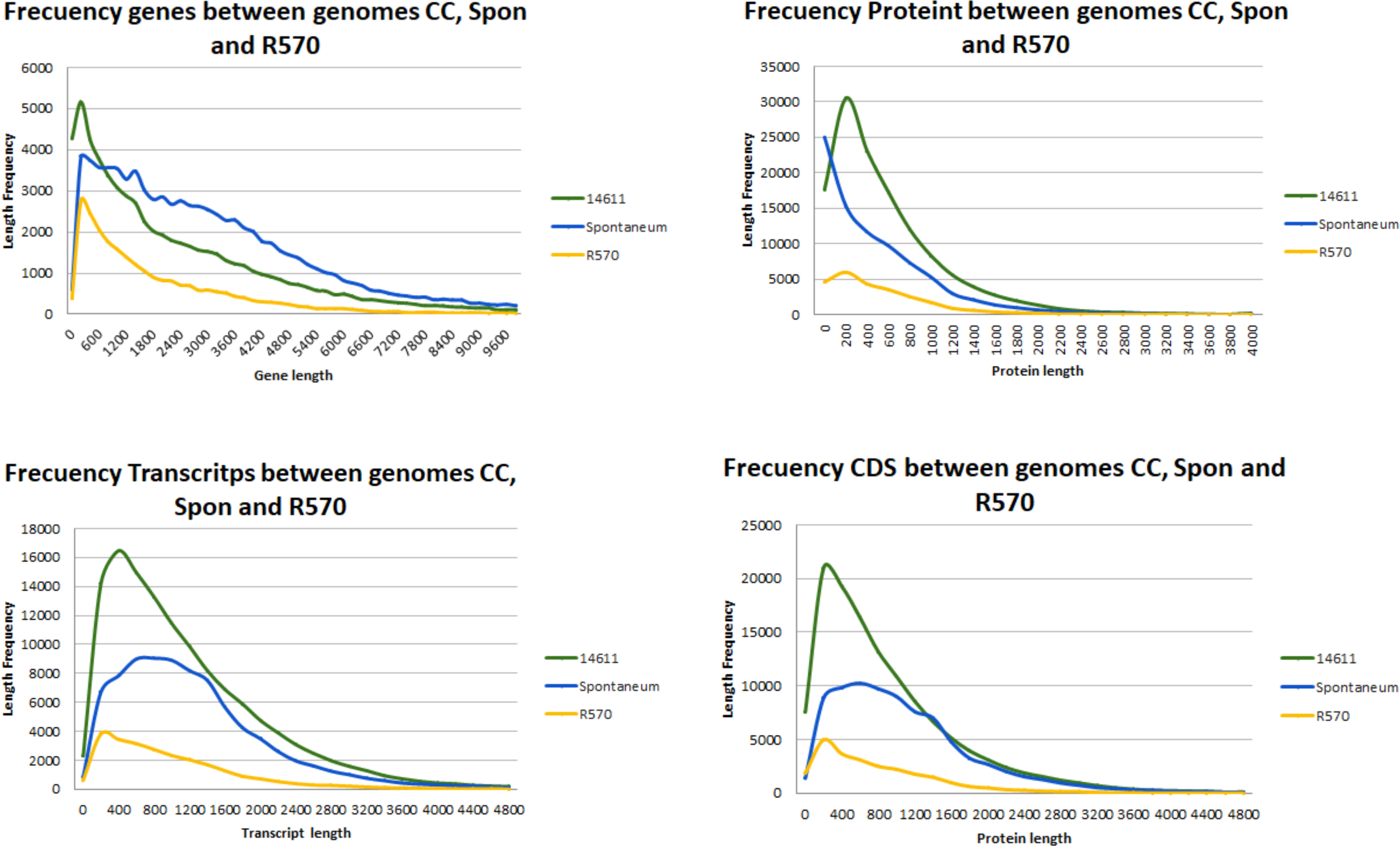

Supplement: Supplementary file 14 [file Image_3.TIFF]

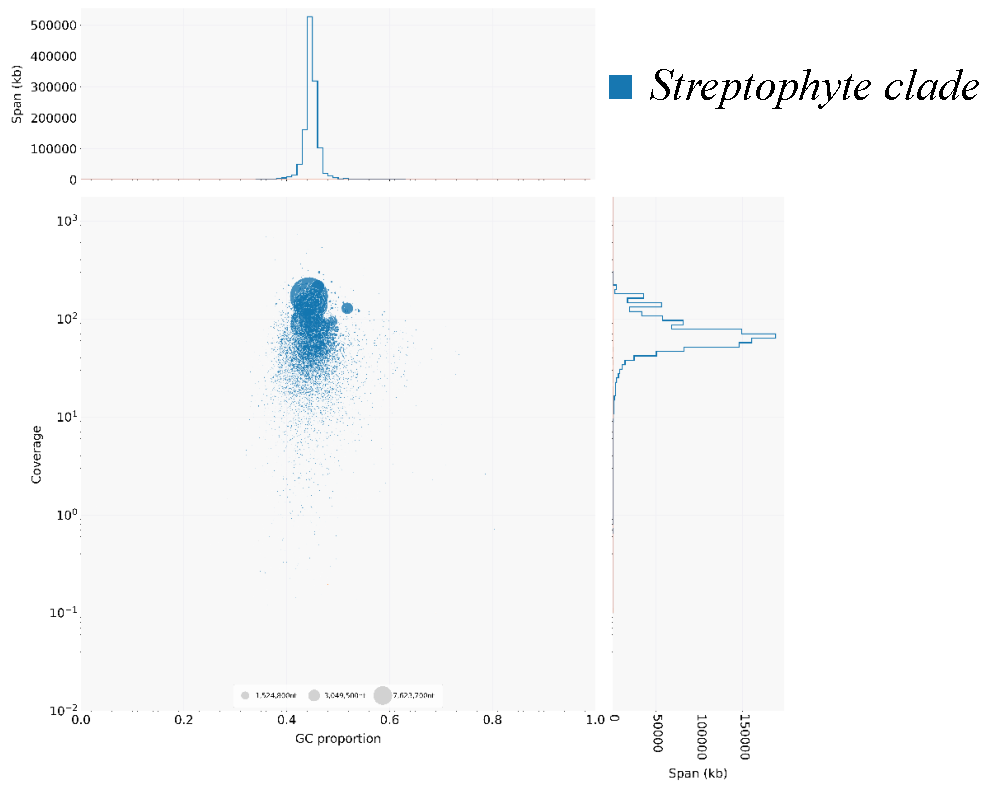

Supplement: Supplementary file 15 [file Image_4.TIF]

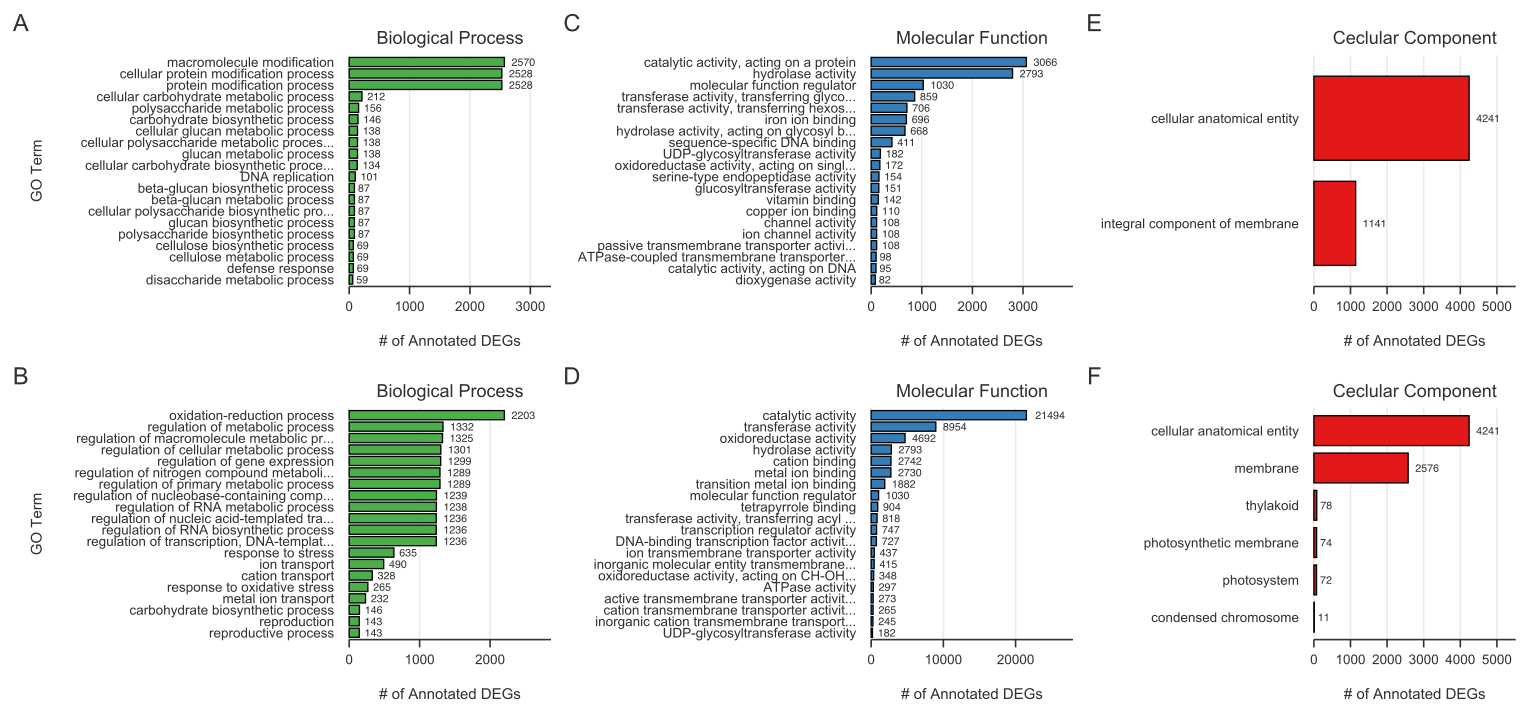

Supplement: Supplementary file 16 [file Image_5.TIFF]
